# Supplementary material for: Association of preterm birth with lipid disorders in early adulthood: A Swedish cohort study
Source: PLoS Med. 2019 Oct 18;16(10):e1002947. doi: 10.1371/journal.pmed.1002947 (PMC6799885; doi:10.1371/journal.pmed.1002947)
Supplement: S1 Appendix — (Table A) Unadjusted HRs for lipid disorders associated with gestational age at birth, stratified by sex, Sweden, 1973 to 2016. (Table B) Adjusted HRs for lipid disorders associated with gestational age at birth, stratified by sex, Sweden, 1973 to 2016. (Table C) Interactions between gestational age at birth and sex in relation to risk of lipid disorders at ages 18 to 44 years. (Table D) Interactions between gestational age at birth and fetal growth in relation to risk of lipid disorders at ages 18 to 44 years. HR, hazard ratio. (DOCX) [file pmed.1002947.s002.docx]

**SUPPORTING INFORMATION**

**Contents**

**Table A.** Unadjusted hazard ratios for lipid disorders associated with Page 2

gestational age at birth, stratified by sex, Sweden, 1973-2016.

**Table B.** Adjusted hazard ratios for lipid disorders associated with Page 3

gestational age at birth, stratified by sex, Sweden, 1973-2016.

**Table C.** Interactions between gestational age at birth and sex in relation Page 4

to risk of lipid disorders at ages 18-44 years.

**Table D.** Interactions between gestational age at birth and fetal growth Page 5

in relation to risk of lipid disorders at ages 18-44 years.

**Table A. Unadjusted hazard ratios for lipid disorders associated with gestational age at birth, stratified by sex, Sweden, 1973-2016.**

|  | **All** | | | | **Men** | | | | **Women** | | | |
| --- | --- | --- | --- | --- | --- | --- | --- | --- | --- | --- | --- | --- |
| **Gestational age at birth** | **Cases** | **Rate^a^** | **HR (95% CI)^b^** | ***P*** | **Cases** | **Rate^a^** | **HR (95% CI)^b^** | ***P*** | **Cases** | **Rate^a^** | **HR (95% CI)^b^** | ***P*** |
| Preterm (<37 wks) | 1,426 | 101.89 | 1.32 (1.25, 1.40) | <0.001 | 910 | 117.33 | 1.29 (1.21, 1.38) | <0.001 | 516 | 82.69 | 1.31 (1.20, 1.44) | <0.001 |
| Extremely preterm (22-27 wks) | 31 | 148.78 | 2.24 (1.57, 3.18) | <0.001 | 15 | 142.13 | 1.85 (1.12, 3.07) | 0.02 | 16 | 155.59 | 2.77 (1.69, 4.52) | <0.001 |
| Very preterm (28-33 wks) | 301 | 111.64 | 1.47 (1.31, 1.64) | <0.001 | 183 | 121.56 | 1.36 (1.17, 1.57) | <0.001 | 118 | 99.10 | 1.59 (1.32, 1.91) | <0.001 |
| Late preterm (34-36 wks) | 1,094 | 98.63 | 1.27 (1.20, 1.36) | <0.001 | 712 | 115.87 | 1.27 (1.18, 1.37) | <0.001 | 382 | 77.22 | 1.22 (1.10, 1.35) | <0.001 |
| Early term (37-38 wks) | 4,187 | 85.99 | 1.15 (1.11, 1.19) | <0.001 | 2,605 | 100.47 | 1.13 (1.08, 1.18) | <0.001 | 1,582 | 69.49 | 1.14 (1.08, 1.20) | <0.001 |
| Full-term (39-41 wks) | 16,712 | 80.12 | Reference |  | 10,032 | 94.37 | Reference |  | 6,680 | 65.31 | Reference |  |
| Post-term (≥42 wks) | 2,725 | 87.04 | 0.96 (0.93, 1.00) | 0.08 | 1,686 | 104.75 | 0.99 (0.94, 1.04) | 0.62 | 1,039 | 68.31 | 0.94 (0.88, 1.00) | 0.06 |
| Per additional week (trend) |  |  | 0.96 (0.95, 0.96) | <0.001 |  |  | 0.96 (0.95, 0.97) | <0.001 |  |  | 0.95 (0.94, 0.96) | <0.001 |

^a^Incidence rate per 100,000 person-years.

^b^Attained age was used as the Cox model time axis.

**Table B. Adjusted hazard ratios for lipid disorders associated with gestational age at birth, stratified by sex, Sweden, 1973-2016.**

|  | **All** | | | | **Men** | | | | **Women** | | | |
| --- | --- | --- | --- | --- | --- | --- | --- | --- | --- | --- | --- | --- |
| **Gestational age at birth** | **Cases** | **Rate^a^** | **HR (95% CI)^b^** | ***P*** | **Cases** | **Rate^a^** | **HR (95% CI)^b^** | ***P*** | **Cases** | **Rate^a^** | **HR (95% CI)^b^** | ***P*** |
| Preterm (<37 wks) | 1,426 | 101.89 | 1.23 (1.16, 1.29) | <0.001 | 910 | 117.33 | 1.22 (1.14, 1.31) | <0.001 | 516 | 82.69 | 1.23 (1.12, 1.34) | <0.001 |
| Extremely preterm (22-27 wks) | 31 | 148.78 | 2.00 (1.41, 2.85) | <0.001 | 15 | 142.13 | 1.68 (1.01, 2.79) | 0.04 | 16 | 155.59 | 2.44 (1.49, 3.98) | <0.001 |
| Very preterm (28-33 wks) | 301 | 111.64 | 1.33 (1.19, 1.49) | <0.001 | 183 | 121.56 | 1.26 (1.09, 1.46) | 0.002 | 118 | 99.10 | 1.46 (1.21, 1.75) | <0.001 |
| Late preterm (34-36 wks) | 1,094 | 98.63 | 1.19 (1.12, 1.26) | <0.001 | 712 | 115.87 | 1.21 (1.12, 1.30) | <0.001 | 382 | 77.22 | 1.15 (1.03, 1.27) | 0.01 |
| Early term (37-38 wks) | 4,187 | 85.99 | 1.09 (1.05, 1.13) | <0.001 | 2,605 | 100.47 | 1.10 (1.05, 1.14) | <0.001 | 1,582 | 69.49 | 1.08 (1.02, 1.14) | 0.01 |
| Full-term (39-41 wks) | 16,712 | 80.12 | Reference |  | 10,032 | 94.37 | Reference |  | 6,680 | 65.31 | Reference |  |
| Post-term (≥42 wks) | 2,725 | 87.04 | 0.99 (0.95, 1.03) | 0.62 | 1,686 | 104.75 | 1.00 (0.95, 1.06) | 0.91 | 1,039 | 68.31 | 0.98 (0.92, 1.04) | 0.50 |
| Per additional week (trend) |  |  | 0.97 (0.96, 0.98) | <0.001 |  |  | 0.97 (0.96, 0.98) | <0.001 |  |  | 0.97 (0.96, 0.98) | <0.001 |

^a^Incidence rate per 100,000 person-years.

^b^Adjusted for child characteristics (birth year, sex, birth order) and maternal characteristics (age, education, birth country or region, BMI, history of lipid disorder).

**Table C. Interactions between gestational age at birth and sex in relation to risk of lipid disorders at ages 18-44 years.**

|  | **Gestational age at birth** | | | | | | HRs (95% CI) for early term vs. full-term within sex strata | HRs (95% CI) for preterm vs. full-term within sex strata |
| --- | --- | --- | --- | --- | --- | --- | --- | --- |
|  | Full-term (39-41 wks) | | Early term (37-38 wks) | | Preterm (<37 wks) | |  |  |
|  | Rate^a^ (Cases) | HR (95% CI)^b^ | Rate^a^ (Cases) | HR (95% CI)^b^ | Rate^a^ (Cases) | HR (95% CI)^b^ |  |  |
| **Sex** |  |  |  |  |  |  |  |  |
| Women | 65.31 (6,680) | Reference | 69.49 (1,582) | 1.10 (1.04, 1.17); *P*<0.001 | 82.69 (516) | 1.25 (1.14, 1.36); *P*<0.001 | 1.10 (1.04, 1.17); *P*<0.001 | 1.25 (1.14, 1.36); *P*<0.001 |
| Men | 94.37 (10,032) | 1.45 (1.40, 1.49); *P*<0.001 | 100.47 (2,605) | 1.57 (1.50, 1.64); *P*<0.001 | 117.33 (910) | 1.76 (1.64, 1.88); *P*<0.001 | 1.08 (1.04, 1.13); *P*<0.001 | 1.21 (1.13, 1.30); *P*<0.001 |
| HRs (95% CI) for men vs. women within gestational age strata |  | 1.45 (1.40, 1.49); *P*<0.001 |  | 1.42 (1.33, 1.51); *P*<0.001 |  | 1.41 (1.26, 1.56); *P*<0.001 |  |  |
| Interaction on additive scale: RERI (95% CI) | | | 0.02 (-0.07, 0.11); *P*=0.68 | | 0.06 (-0.10, 0.23); *P*=0.43 | |  |  |
| Interaction on multiplicative scale: HR ratio (95% CI) | | | 0.98 (0.91, 1.05); *P*=0.63 | | 0.98 (0.87, 1.09); *P*=0.66 | |  |  |

^a^Incidence rate per 100,000 person-years.

^b^Adjusted for child characteristics (birth year, birth order) and maternal characteristics (age, education, birth country or region, BMI, history of lipid disorder).

HR = hazard ratio, RERI = relative excess risk due to interaction

**Table D. Interactions between gestational age at birth and fetal growth in relation to risk of lipid disorders at ages 18-44 years.**

|  | **Gestational age at birth** | | | | | | HRs (95% CI) for early term vs. full-term within fetal growth strata | HRs (95% CI) for preterm vs. full-term within fetal growth strata |
| --- | --- | --- | --- | --- | --- | --- | --- | --- |
|  | Full-term (39-41 wks) | | Early term (37-38 wks) | | Preterm (<37 wks) | |  |  |
|  | Rate^a^ (Cases) | HR (95% CI)^b^ | Rate^a^ (Cases) | HR (95% CI)^b^ | Rate^a^ (Cases) | HR (95% CI)^b^ |  |  |
| **Fetal growth** |  |  |  |  |  |  |  |  |
| AGA | 77.91 (13,027) | Reference | 83.00 (3,289) | 1.08 (1.04, 1.12); *P*<0.001 | 99.65 (1,137) | 1.24 (1.17, 1.32); *P*<0.001 | 1.08 (1.04, 1.12); *P*<0.001 | 1.24 (1.17, 1.32); *P*<0.001 |
| SGA | 107.41 (2,284) | 1.27 (1.21, 1.33); *P*<0.001 | 129.49 (459) | 1.59 (1.45, 1.75); *P*<0.001 | 121.30 (157) | 1.50 (1.28, 1.76); *P*<0.001 | 1.26 (1.13, 1.38); *P*<0.001 | 1.19 (0.99, 1.38); *P*=0.06 |
| HRs (95% CI) for SGA vs. AGA within gestational age strata |  | 1.27 (1.21, 1.33); *P*<0.001 |  | 1.47 (1.33, 1.62); *P*<0.001 |  | 1.21 (1.01, 1.41); *P*=0.04 |  |  |
| Interaction on additive scale: RERI (95% CI) | | | 0.24 (0.08, 0.40); *P*=0.003 | | -0.01 (-0.26, 0.25); *P*=0.97 | |  |  |
| Interaction on multiplicative scale: HR ratio (95% CI) | | | 1.16 (1.04, 1.29); *P*=0.01 | | 0.96 (0.79, 1.12); *P*=0.60 | |  |  |

^a^Incidence rate per 100,000 person-years.

^b^Adjusted for child characteristics (birth year, sex, birth order) and maternal characteristics (age, education, birth country or region, BMI, history of lipid disorder).

AGA = appropriate for gestational age, HR = hazard ratio, RERI = relative excess risk due to interaction, SGA = small for gestational age
