# Supplementary material for: Association of preterm birth with lipid disorders in early adulthood: A Swedish cohort study
Source: PLoS Med. 2019 Oct 18;16(10):e1002947. doi: 10.1371/journal.pmed.1002947 (PMC6799885; doi:10.1371/journal.pmed.1002947)
Supplement: Attachment — Submitted filename: crump.pdf [file pmed.1002947.s003.pdf]

This paper describes an analysis using routine data–sets of the relationship between pre–term birth and lipid disorders in early adulthood.

I was asked for a statistical report and I interpret that to include all aspects of the design and conduct of the study.

Points of detail

Title Perhaps early adulthood might be better? On average they were about 30 years old.

Page 7 With only 1% missing multiple imputation hardly seems necessary so I assume the complete cases were in fact fewer than 99%. Did the authors do a complete cases analysis as a sensitivity analysis?

Page 7 I think the fact that proportional hazards was met belongs in the results along with any other checks.

Page 8 I have the feeling that this sort of model is usually called a frailty model rather than a stratified model which I would have thought was rather different.

Point of more substance

In the abstract the authors state that the co–sibling analysis suggested that their findings were '... partially explained by ...'. On page 12 they state '... substantially though not completely explained ...'. If we look at the figures for preterm birth (which is probably the more precisely estimated) it goes from 1.23 down to 1.10. To my mind page 12 is the better wording as it does represent about a halving of the relative risk. Incidentally I agree with the authors' decision not to place any weight on the shift from above to below some arbitrary level of statistical significance.

Summary

No major issues from me.

Michael Dewey
