# Supplementary material for: Association of preterm birth with lipid disorders in early adulthood: A Swedish cohort study
Source: PLoS Med. 2019 Oct 18;16(10):e1002947. doi: 10.1371/journal.pmed.1002947 (PMC6799885; doi:10.1371/journal.pmed.1002947)
Supplement: Attachment — Submitted filename: Crump_Response_to_Reviewers_PLOS_Med.docx [file pmed.1002947.s004.docx]

August 12, 2019

Dr. Casey Crump

Icahn School of Medicine at Mount Sinai

One Gustave L. Levy Place, Box 1077

New York, NY 10029

RE: PMEDICINE-D-19-01959

Dear Editor and Reviewers:

Thank you for your favorable review of our manuscript entitled “Preterm Birth and Risk of Lipid Disorders in Adulthood: A National Cohort Study”. We appreciate your many positive comments, e.g., “Overall, the paper is presenting convincing research results and I believe it is a good candidate for being published in PLOS Medicine… The paper is well written… The research presented in the paper is of good quality and is scientifically sound.” We also appreciate your helpful suggestions for strengthening the paper further. We are now submitting a revised manuscript for your consideration after responding to each of those suggestions. Our detailed responses are listed below.

**EDITOR COMMENTS**

1) “Abstract – please state which country this is based in and also in title.”

We have added “Swedish” to both the title and Abstract (“Methods and Findings” section, 1^st^ sentence).

2) “Abstract – Please add demographic details and include a sentence on the limitations of the study as the final sentence of the ‘methods and Findings section’. Please include p values for all 95%CIs (and in the main text). ‘these findings were similar in men and women’ – please be accurate; similar is ambiguous.”

We have now added demographic information (% women) in the Abstract (“Methods and Findings” section, 1^st^ sentence), as well as the limitation that we lacked information on specific types or severity of lipid disorders (Abstract, “Methods and Findings” section, last sentence). We also have added *P* values in the Abstract (“Methods and Findings” section, 5^th^ sentence) and throughout the paper, and provided more details on risk estimates among men and women (Abstract, “Methods and Findings” section, 6^th^ sentence).

3) “Data – you mention that some restrictions will apply, then you say all data is available. Can you please clarify, with the caveat that PLOS has a data availability policy. PLOS Medicine requires that the de-identified data underlying the specific results in a published article be made available, without restrictions on access, in a public repository or as Supporting Information at the time of article publication, provided it is legal and ethical to do so. Please see the policy at [https://urldefense.proofpoint.com/v2/url?u=http-3A__journals.plos.org_plosmedicine_s_data-2Davailability&d=DwIGaQ&c=shNJtf5dKgNcPZ6Yh64b-A&r=whWIjs1wXHLkBihLFGBKw9ffghgusV_IhHagryE5ycs&m=Qs7Xmiej3Z4Q_OP681sm-YEujP5oIIipIDLxgasefWI&s=sP95ghANGE30ANnkTy26mqlnMaBDaS3GW0SnG2oIspg&e=](https://mshmsvpn.mssm.edu/f5-w-68747470733a2f2f75726c646566656e73652e70726f6f66706f696e742e636f6d$$/v2/url?u=http-3A__journals.plos.org_plosmedicine_s_data-2Davailability&d=DwIGaQ&c=shNJtf5dKgNcPZ6Yh64b-A&r=whWIjs1wXHLkBihLFGBKw9ffghgusV_IhHagryE5ycs&m=Qs7Xmiej3Z4Q_OP681sm-YEujP5oIIipIDLxgasefWI&s=sP95ghANGE30ANnkTy26mqlnMaBDaS3GW0SnG2oIspg&e=) and FAQs at [https://urldefense.proofpoint.com/v2/url?u=http-3A__journals.plos.org_plosmedicine_s_data-2Davailability-23loc-2Dfaqs-2Dfor-2Ddata-2Dpolicy&d=DwIGaQ&c=shNJtf5dKgNcPZ6Yh64b-A&r=whWIjs1wXHLkBihLFGBKw9ffghgusV_IhHagryE5ycs&m=Qs7Xmiej3Z4Q_OP681sm-YEujP5oIIipIDLxgasefWI&s=pBEEd6jdo2Trls_STNZbup8ZQZ1dLC7QtcsO-voJsro&e=](https://mshmsvpn.mssm.edu/f5-w-68747470733a2f2f75726c646566656e73652e70726f6f66706f696e742e636f6d$$/v2/url?u=http-3A__journals.plos.org_plosmedicine_s_data-2Davailability-23loc-2Dfaqs-2Dfor-2Ddata-2Dpolicy&d=DwIGaQ&c=shNJtf5dKgNcPZ6Yh64b-A&r=whWIjs1wXHLkBihLFGBKw9ffghgusV_IhHagryE5ycs&m=Qs7Xmiej3Z4Q_OP681sm-YEujP5oIIipIDLxgasefWI&s=pBEEd6jdo2Trls_STNZbup8ZQZ1dLC7QtcsO-voJsro&e=)”

We have clarified that the national registry data on which this study was based were analyzed under strict confidentiality agreements with Swedish authorities. Due to ethical and legal concerns, the supporting data (which come from a large portion of the Swedish population) cannot be made openly available. Further information about the data registries is available from the Swedish National Board of Health and Welfare: <https://www.socialstyrelsen.se/en/statistics-and-data/registers/>. Researchers are also welcome to contact the authors of this paper if any questions arise.

4) “At this stage, we ask that you include a short, non-technical Author Summary of your research to make findings accessible to a wide audience that includes both scientists and non-scientists. The Author Summary should immediately follow the Abstract in your revised manuscript. This text is subject to editorial change and should be distinct from the scientific abstract. Please see our author guidelines for more information: [https://urldefense.proofpoint.com/v2/url?u=https-3A__journals.plos.org_plosmedicine_s_revising-2Dyour-2Dmanuscript-23loc-2Dauthor-2Dsummary&d=DwIGaQ&c=shNJtf5dKgNcPZ6Yh64b-A&r=whWIjs1wXHLkBihLFGBKw9ffghgusV_IhHagryE5ycs&m=Qs7Xmiej3Z4Q_OP681sm-YEujP5oIIipIDLxgasefWI&s=6-kA5rgXLIQxmbM0Ytb-ypn88IN31jHw2yEva5SE_00&e=](https://mshmsvpn.mssm.edu/f5-w-68747470733a2f2f75726c646566656e73652e70726f6f66706f696e742e636f6d$$/v2/url?u=https-3A__journals.plos.org_plosmedicine_s_revising-2Dyour-2Dmanuscript-23loc-2Dauthor-2Dsummary&d=DwIGaQ&c=shNJtf5dKgNcPZ6Yh64b-A&r=whWIjs1wXHLkBihLFGBKw9ffghgusV_IhHagryE5ycs&m=Qs7Xmiej3Z4Q_OP681sm-YEujP5oIIipIDLxgasefWI&s=6-kA5rgXLIQxmbM0Ytb-ypn88IN31jHw2yEva5SE_00&e=)”

We have now provided an Author Summary (page 4, immediately after Abstract).

5) “"was associated" at line 303; "need" seems too strong at lines 41 and 309”

We have made these changes accordingly (Discussion, last paragraph, 1^st^ sentence; Abstract, last paragraph, last sentence; and Discussion, last paragraph, last sentence).

6) “remove page numbers from STROBE checklist – this should be sections and paragraphs, as they can change during page layout.”

We have replaced page numbers with sections and paragraphs in a newly revised STROBE checklist.

7) “Causal language – Line 274 – “have several clinical implications” as this is not a trial and an observational study, please tone down causal, overheated language – for example, ‘may have’; also Line 275 “should” replace with ‘may’ and again “should be recognized as a risk factor”…’may be taken into account when considering’ ….Please adjust throughout where such language occurs.”

We have softened the language here and elsewhere throughout the text (e.g., Discussion, 5^th^ paragraph, 2^nd^ sentence; last paragraph, 3^rd^-4^th^ sentences).

**REVIEWER COMMENTS**

**Reviewer #1**

“This paper describes an analysis using routine datasets of the relationship between preterm birth and lipid disorders in early adulthood. I was asked for a statistical report and I interpret that to include all aspects of the design and conduct of the study.”

1) “Title Perhaps early adulthood might be better? On average they were about 30 years old.”

We have added the word “early” to the title.

2) “Page 7 With only 1% missing multiple imputation hardly seems necessary so I assume the complete cases were in fact fewer than 99%. Did the authors do a complete cases analysis as a sensitivity analysis?”

Data were highly complete for all variables except maternal BMI, which was ascertained at the beginning of prenatal care starting in 1982 and was available for 38.8% of women. We have now performed a complete case analysis as an alternative to multiple imputation. All risk estimates were negligibly changed from those of the main analysis (e.g., preterm vs. full-term, fully adjusted HR: 1.23; 95% CI, 1.08-1.39; *P*=0.002). We now report this sensitivity analysis in the Methods (“Statistical Analysis” section, 4^th^ paragraph, 5^th^ sentence) and Results (“Other Sensitivity Analyses” section, 2^nd^ paragraph).

3) “Page 7 I think the fact that proportional hazards was met belongs in the results along with any other checks.”

Assessment of the proportional hazards assumption has now been moved to the Results (“Associations Between Gestational Age at Birth and Lipid Disorders” section, 3^rd^ paragraph, last sentence).

4) “Page 8 I have the feeling that this sort of model is usually called a frailty model rather than a stratified model which I would have thought was rather different.”

We used a stratified Cox model rather than a shared-frailty model. The stratified Cox model was stratified on sets of siblings defined by the same biological mother, which allows the baseline hazard function to differ across families, thus accounting for their shared genetic and/or environmental factors. Both the stratified and shared-frailty models are considered valid approaches to account for within-group correlation in survival time data. However, the stratified Cox model is computationally much simpler and more feasible than the shared-frailty model, which frequently encounters matrix-size limitations and convergence problems, especially with large datasets as in the present study. The stratified Cox model can result in less precision but this is negligible with large sample sizes as in this study.

5) “In the abstract the authors state that the co-sibling analysis suggested that their findings were `. . . partially explained by . . . '. On page 12 they state `. . . substantially though not completely explained . . . '. If we look at the figures for preterm birth (which is probably the more precisely estimated) it goes from 1.23 down to 1.10. To my mind page 12 is the better wording as it does represent about a halving of the relative risk. Incidentally I agree with the authors' decision not to place any weight on the shift from above to below some arbitrary level of statistical significance.”

We agree and have now changed this wording accordingly in the Abstract (“Methods and Findings” section, 7^th^ sentence).

6) “Summary: No major issues from me.”

We appreciate your helpful comments, which we feel have further improved the paper.

**Reviewer #2**

“The authors examined longitudinal associations of preterm birth with a risk of developing lipid disorders in young adulthood (age 18-44). This study is unique and highlighting the potential of shaping a national database into a birth cohort for research on adult-onset diseases. However, the strength of clinical implications was challenging to judge, and there are several concerns. The reviewer is providing main and minor comments hereafter.”

“Major comments:”

1) “The authors defined "lipid disorders" using ICD codes. The codes included ICD-8/9: 272 and ICD-10: E66.

The latter includes the followings:

E66 Overweight and obesity

- E66.0 Obesity due to excess calories

- E66.1 Drug-induced obesity

- E66.2 Morbid (severe) obesity with alveolar hypoventilation

- E66.3 Overweight

- E66.8 Other obesity

- E66.9 Obesity, unspecified

ICD-10 E66 is for obesity or overweight, not for lipid disorders. It may be uncommon for adults <44 years go to a clinic just because they are overweight or obese. So, the observed low risk of being recorded with "E66" would make sense. The authors should check back what information the authors extracted. If the authors had used E66 literally, the authors should revise it to study lipid disorders as the authors aimed and redo all the analysis.”

We appreciate your pointing this out and apologize for this error. “E66” was listed in the text inadvertently, and instead should have said “E78” (lipid disorders). We have confirmed that E78 was the *ICD*-10 diagnostic code used in the data extraction and all analyses. The large majority (>80%) of these diagnoses were not further specified, and therefore it was not possible to analyze more specific types of lipid disorders. We have now corrected the *ICD*-10 diagnostic code reported in the Methods (“Ascertainment of Gestational Age at Birth and Lipid Disorders” section, 2^nd^ paragraph, 2^nd^ sentence).

2) “The validity of the longitudinal analysis is unclear or not identifiable. The authors evaluated lipid disorders at ages 18-44 years as "lipid disorders in adulthood". Therefore, the authors should not count any lipid disorders before participants became 18 years old. Also, there is a possibility that a lipid disorder occurred during a fetal period and caused a preterm delivery. It is not clear how the authors treated those individuals who developed such a fetal, infantile, or juvenile lipid disorder.

The authors are describing that one of the inclusion criteria for this cohort was "still living in Sweden at age 18 years". This description did not rule out the possibility that entry of adolescents with a lipid disorder into the cohort.

The authors should confirm and clarify that the authors had evaluated participants who were free from a lipid disorder at the age of 18 years. If the authors were not sure, the authors should do additional analyses, for example, excluding those who were treated at least once with lipid-lowering drugs during 18-20 years of age.

Sipola-Leppanen et al. reported the association of preterm delivery with unfavourable lipid profiles in adolescents (Pediatrics, 2014;134(4):e1072-81). Other studies exist sufficiently to raise the concern about censoring and the risk set in this study.”

We appreciate this comment and have now clarified that the study population included all persons born as singletons in Sweden during 1973-1995 who were still living in Sweden at age 18 years *without a prior diagnosis of lipid disorders*. This approach enabled assessment of newly diagnosed lipid disorders in adulthood. All persons diagnosed with lipid disorders prior to age 18 years (n=1,162) were therefore excluded (Methods, “Study Population” section, 1^st^ paragraph, 4^th^ sentence).

3) “The clinical relevance may be much weaker than the extent which the authors are describing. This point may be crucial and should not be discussed just as a limitation or weakness in the Discussion. Analysis should deal with it.

We should note that lipid disorders in this manuscript were those developed before the age of 44 years as the primary outcome. The average incidence rate was approximately 0.1% (100/100000) (Table 2). The clinical implications for such a rare event may require the focus on many possible rare conditions in childhood.

The concern is about secondary dyslipidemia. Many children or young adults may have experienced an onset of respiratory disease, dermatological disease, an endocrine disorder, or another. They might receive medications which influence lipid homeostasis and induce secondary dyslipidemia (e.g. Henkin et al., Secondary Dyslipidemia Inadvertent Effects of Drugs in Clinical Practice, JAMA, 1992;267(7):961-968).

For participants treated with other medications for their primary disease, lipid profiles are subject to monitoring and treatment. For clinicians who often face secondary dyslipidemia anyhow, this paper may not be informative, and the authors may not need to highlight the importance.

This point partly relates to the comment above about a lipid disorder or any other diseases arising before aged 18 years. The authors should account for this plausibility over the course from the analysis stage (see the other comments) to the interpretation and discussion.

The authors discuss clinical implications on Page 13. This part may represent what paediatricians already know. Birth outcomes, including preterm delivery, may influence a child-onset disease that requires a particular medication and the medical condition further influences lipid profiles and further cardiovascular health in later life. If that is a causal pathway, the authors' implication would require revision.

In addition to secondary dyslipidemia, preterm birth may relate to a growth spurt and later obesity or overweight. Concerning the obesity issue, physicians commonly pay attention to preterm birth and childhood obesity. Then, lipid disorders and other cardiometabolic outcomes may get concerning reasonably. Regarding many correlated issues and causal pathways, clinicians may have already recognised the importance of preterm delivery for cardiovascular risk factors, not only lipid disorders. From this broad view, again, the clinical implication of this study seems to be weak. The authors need to account for related, well-known issues which pediatricians have already know.”

We appreciate these comments and have now provided a sensitivity analysis to examine the potential influence of lipid disorders that were secondary to medications. These analyses explored medications that have been most strongly associated with lipid disorders (e.g., corticosteroids, antiepileptics, phenothiazines, androgens, cyclosporine, and retinoid medications for systemic use). All newly diagnosed lipid disorders that were preceded by at least 2 prescriptions of any of these medications were considered to be potentially secondary lipid disorders. Among all persons diagnosed with lipid disorders, 354 (25.6%) of those born preterm and 4,098 (25.1%) of those born full-term met this definition. After repeating the analyses while alternately including or excluding these cases as part of the study outcome, all results were negligibly changed. For example, when including them, the fully adjusted HR for lipid disorders associated with preterm birth was 1.22 (95% CI, 1.15-1.29; *P*<0.001), compared to 1.23 (95% CI, 1.16-1.32; *P*<0.001) after excluding them. The corresponding HRs associated with extremely preterm birth were 1.91 (95% CI, 1.31-2.79; *P*=0.001) and 1.99 (95% CI, 1.28-3.08; *P*=0.002), respectively. These findings suggest that secondary lipid disorders had little influence on the overall findings. These new sensitivity analyses are now reported in the Methods (“Statistical Analyses” section, 4^th^ paragraph, 2^nd^-4^th^ sentences) and Results (“Other Sensitivity Analyses” section, 1^st^ paragraph).

4) “The authors state the availability of primary and secondary diagnoses (Line 94-108). The authors seem to be able to identify different types of medications, not only lipid-lowering medications.

Therefore, the authors should address the concern of secondary dyslipidemia above raised. Note that some observational studies using an extensive medical database have incorporated as much information as possible, including different types of medications and clinical diagnoses (e.g. Hippisley-Cox et al., BMJ, 2017;357:j2099). The similar effort would be crucial.”

We agree and, as noted above (Comment #4), have now assessed the potential influence of lipid disorders that were secondary to 6 different categories of medications that are most strongly associated with lipid disorders, including commonly used medications such as corticosteroids, antiepileptics, and phenothiazines. We found that excluding such cases of lipid disorders had a negligible effect on the overall findings (Methods, “Statistical Analyses” section, 4^th^ paragraph, 2^nd^-4^th^ sentences; Results, “Other Sensitivity Analyses” section, 1^st^ paragraph).

5) “Co-sibling analyses are unique and informative for readers. Future readers will be careful of the potential of using a national database containing similar types of information.

The authors described that the sample size of the co-sibling analysis was approximately 80% of the total number of participants. The authors should explain or revise those participants fully contributed to the co-sibling analysis. For example, if one family has two siblings, and if both did not develop lipid disorders, the within-family matched analysis would not use them. With the rarity of the events, effective sample sizes of the co-sibling analyses might be much smaller than those presented. The authors should verify and clarify it. (note: the continuous estimate in Table 3 had nearly five times greater variance than that in Table 2.)

The authors’ interpretation from the co-sibling analyses seems to be valid, but at the same time, the authors may want to describe the nature of the uncertainty as well.”

We appreciate these comments. In the co-sibling analyses using Cox regression, there is essentially no concordance of outcomes among siblings, because the outcome is not binary (lipid disorders vs. not) but instead “time-to-event” (i.e., time to the date of diagnosis of lipid disorders, with censoring at death or emigration). All individuals in the study cohort who had siblings contributed to these analyses. The stratified Cox model accounts for correlation due to their shared exposures by allowing a separate baseline hazard function for each family. These analyses yield wider 95% CIs than the main analyses because of the exclusion of individuals without siblings as well as accounting for potential correlation within families. We have now clarified that 1,881,473 individuals in 846,164 families had at least one sibling and were included in these analyses (Methods, “Statistical Analysis” section, 2^nd^ paragraph, 3^rd^ sentence).

“Minor comments:”

6) “Table 2 and Table 3 do not need to show the results after sex-stratification. The results by sex were unremarkable. There is no clear, strong rationale or hypothesis to indicate the effect modification by sex. Also, equivalent and additional pieces of information are available in the supplementary materials.”

We have now removed the sex-stratified results from Tables 2 and 3.

7) “In both Table 2 and Table 3, the authors should include hazard ratios (95% confidence intervals) from different regression models. Unadjusted HRs are worth presenting. Changes in HRs via different adjustments could be meaningful to present (e.g. adjustment for child characteristics with or without adjustment for maternal characteristics). "Preterm or not" showed unadjusted HR=1.32 and adjusted HR=1.23. The shift is informative about mechanisms of the complex association of early-life environment with lipid disorders recorded in later life.”

We agree and have now presented results from three models in Table 2: unadjusted, “adjusted for child characteristics”, and “adjusted for child and maternal characteristics”. In Table 3, we present results from co-sibling analyses that alternately were not further adjusted, then further adjusted for child and maternal characteristics. (Adjusting the co-sibling analyses first for child characteristics and then additionally for maternal characteristics resulted in a negligible change in risk estimates.)

8) “For the analysis treating gestational age as a continuous variable, the authors may better use hazard ratio per 5 weeks. One week is of a too narrow range given the distribution of the gestational ages, and the point estimates were not informative. The point estimates and 95% CIs in Table 2 and Table 3 were informative enough for some readers to calculate p-values. The authors may better avoid inconsistent presentation between Table 2 and Table 3 (i.e. p-values, which the authors may delete from Table 2).”

We have now modeled gestational age as a continuous variable in units of “per 5 weeks” (Tables 2 and 3), and have updated the results accordingly in the Abstract and Results. We are open to removing *P* values from Tables 2 and 3, although the Editor requested that *P* values be included with all 95% CI in the text (we are unclear if this should include the tables as well). We so far have kept *P* values in the tables but will leave this to the Editor’s discretion.

9) “Methods: The reviewer wonders if the information on C-section was available.”

Yes, C-section information is available. We have now explored interactions between gestational age and mode of delivery (C-section vs. vaginal) in relation to the risk of lipid disorders. We found that the association between preterm birth and risk of lipid disorders was slightly stronger among persons delivered by C-section (adjusted HR, 1.32; 95% CI, 1.18-1.48; N=225,315 [10.1% of the cohort]) than among those delivered vaginally (1.18; 95% CI, 1.11-1.26; N=2,009,469 [89.9%]) (tests for interaction: additive, *P*=0.06; multiplicative, *P*=0.09). This secondary analysis is now reported in the Methods (“Statistical Analyses” section, 4^th^ paragraph, 1^st^ sentence) and Results (“Interactions” section, 3^rd^ paragraph).

10) “Results: Some text, Page 10 in particular, was hard to read. Readers expect to read results, not descriptions of the methods, rationales, and interpretations. Those should be in Methods or Discussion. The authors should document what results were from which analysis.”

We have now edited this section accordingly to improve readability. Specifically, we moved all the descriptions of co-sibling sensitivity analyses to the Methods (“Statistical Analysis” section, new 3^rd^ paragraph) and the interpretation of these analyses to the Discussion (3^rd^ paragraph, last sentence).

11) “The results of the co-sibling analyses should include information on the number of families, not only individual adults.”

We have now clarified that these analyses included 1,881,473 individuals from 846,164 families (Methods, “Statistical Analysis” section, 2^nd^ paragraph, 3^rd^ sentence).

12) “Line 201-204: The text seems that the authors are obsessed with statistical significance. "slightly lower" is just fine with p for interaction = 0.41. Not wrong. The next sentence should be in the Discussion section.”

We have now changed this wording to “slightly lower” and removed the reference to statistical significance (Results, “Co-Sibling Analyses” section, 2^nd^ paragraph, 4^th^ sentence). We also moved the subsequent sentence to the Discussion (3^rd^ paragraph, last sentence).

13) “Line 206-213: The authors should note the analysis of within-family carryover effects in Methods. Then, the sample size should be clarified. This analysis must have included families with discordant outcomes in their siblings only. The authors should not use the phrase "case-crossover" as it is confusing and should correspond to a particular study design which the authors did not adopt.”

We have moved the description of these analyses to the Methods, provided the relevant sample sizes, and removed the term “case-crossover” (Methods, “Statistical Analysis” section, 3^rd^ paragraph, 4^th^ sentence; Results, “Co-Sibling Analyses” section, 2^nd^ paragraph, 3^rd^ sentence).

14) “In the Discussion, the authors cited information on genetic factors influencing both timing of delivery and lipid disorder in an offspring. The authors bind this to the finding just once in the Discussion.”

Although the underlying mechanisms are still unknown, we have discussed the possibility that shared genetic factors could potentially explain the association we found between preterm birth and lipid disorders. We also have further highlighted that future studies of shared genetic factors that influence both the timing of birth and lipid metabolism may help elucidate the specific mechanisms (Discussion, last paragraph, 3^rd^ sentence).

“Figures/Tables:”

15) “Figure 1 should not include the label of 0.00 as 0.00 is not possible. Also, the authors should provide each point estimate and 95% CI on the right of the panel. The horizontal axis covers a too wide range of HRs, and then uncertainty (CIs) give a false impression of narrow ones. Present the range up to 2.5 rather than 3.0 and use an arrow to the right.”

We have now revised Figure 1 accordingly. Specifically, we changed the horizontal axis to range from 0.5 to 2.5, and reported the HRs and 95% CIs in the right panel (Figure 1).

**Reviewer #3**

“This work reveal important associations between how gestational lengths may effect serious complications later in life, lipid disorders, which is the underlying cause of cardiovascular diseases in adults. This knowledge is important for understanding how diseases arise and but could also be important for clinicians to recognize early risk factors in patients and encourage life style changes in reducing the risk of diseases to improve public health.

The paper is well written. The research presented in the paper is of good quality and is scientifically sound. I have only some minor comments:”

1) “In the abstract line 2, it would be interesting to know what type of disorders you are referring to, if word counts accept this.”

We lacked information on specific types of lipid disorders, but they likely included the most common lipid disorders in the general population and in prior smaller studies of preterm birth (e.g., elevated LDL cholesterol or triglycerides). We have now clarified this in the Abstract (“Background” section, 1^st^ sentence).

2) “Line 26, comment on type of cohort study, a retrospective design?”

We have added the word “retrospective” (i.e., data collection was completed prior to conducting this study) (Abstract, “Methods and Findings” section, 1^st^ sentence).

3) “Line 27, why did you choose this time period? Many cases are still young. Would it be easier to see associations in older age groups? Later in the manuscript I did understand that you choose this period because of the registry and no data was accessible before this time period. If you had access to data from older age groups, how do you think this would have affected the results?”

We included persons born from 1973 (inception of the Swedish Birth Registry) through 1995, in order to have sufficient follow-up into adulthood. We have clarified these points in the Methods (“Study Population” section, 1^st^ paragraph, 1^st^-3^rd^ sentences). Because the biggest advances in neonatal care occurred in the 1970s-80s (e.g., surfactant, antenatal corticosteroids, mechanical ventilation), the earliest birth cohorts who survived due to those advances are now in their 40s. Consequently, it is not yet possible to examine lipid disorders at older ages in large cohorts of preterm survivors, and it remains unknown how such associations would compare with the findings of the present study. We indicate in the limitations section that additional follow-up will be needed to examine this outcome in later adulthood when such data become available (Discussion, 7^th^ paragraph, 7^th^ sentence).

4) “Line 34, why did you use full-term birth only gestational week 39-41?”

We have clarified that early term birth (37-38 weeks) was examined as a separate category because it has previously been associated with increased cardiovascular- and endocrine-related mortality relative to later term birth. As a consequence, the reference category was full-term birth, defined as gestational ages 39-41 weeks. This is also consistent with most recent studies of preterm birth and with definitions recommended by the American College of Obstetrics and Gynecology (Methods, “Ascertainment of Gestational Age at Birth and Lipid Disorders” section, 1^st^ paragraph, 3^rd^ sentence).

5) “Line 50, again a short introduction about what type of lipid disorders you are referring to.”

We have now clarified that this refers to the most common lipid disorders in the general population, such as elevated LDL cholesterol or triglycerides (Introduction, 1^st^ paragraph, 4^th^ sentence).

6) “Line 86, gestational length is collected from both last menstruation period and ultrasound. How do you this this affects the results in the study?”

We assessed this by performing a sensitivity analysis restricted to persons born in 1982 or later, when gestational ages were determined predominantly by ultrasound. Compared to the main analysis, the risk estimates were minimally changed (e.g., preterm vs. full-term, fully adjusted HR: 1.25; 95% CI, 1.14-1.37; *P*<0.001), suggesting that differences in the method of estimating gestational age were unlikely to have a major influence on the findings. We now briefly summarize these findings in the Methods (“Statistical Analysis” section, 4^th^ paragraph, 6^th^ sentence) and Results (“Other Sensitivity Analyses” section, 2^nd^ paragraph, last sentence).

7) “Line 114. Some covariates are missing e.g. smoking, physical activity and diet. How do you think this would impact the results if they were available?”

Lifestyle factors such as smoking, physical activity, and diet could potentially be relevant either (1) in the mothers (i.e., prior to birth of the offspring) or (2) in the offspring later in life. Regarding the former, our co-sibling analyses controlled for shared familial factors, which may include maternal behavioral factors such as smoking, physical activity, or diet that could potentially contribute to both preterm delivery and the development of lipid disorders in the offspring. Regarding the latter, behavioral factors in the offspring later in life are potential modifiers rather than confounders of the association between preterm birth and the development of lipid disorders. It seems likely that persons born prematurely could modify their risk of lipid disorders through healthy behaviors later in life, though to what extent remains to be demonstrated. Additional assessment of behavioral risk factors later in life would be useful to further elucidate potential modifiers of the association between preterm birth and lipid disorders in future studies with access to this information. We have now clarified these points in the Discussion (7^th^ paragraph, 5^th^-6^th^ sentences).

8) “In the discussion part you are presenting some studies that has looked at specific lipids as for example triglycerides. Triglycerides are more of a risk factor for CVD in women. Was it not possible to analyze from the Registry?”

The large majority (>80%) of lipid disorder diagnoses were not further specified, and thus information was unavailable in sufficient numbers to analyze specific types of lipid disorders. We have further clarified this limitation both in the Abstract (“Methods and Findings” section, last sentence) and Discussion (7^th^ paragraph, 3^rd^ sentence).

9) “Why did you choose Cox regression model?”

A Cox model made most efficient use of the available data, including detailed information on date of diagnosis of lipid disorders. In addition, model diagnostics (e.g., log-log plots to assess the proportional hazards assumption) showed a good fit of these models (Results, “Associations Between Gestational Age at Birth and Lipid Disorders” section, 3^rd^ paragraph, last sentence).

10) “Overall, the paper is presenting convincing research results and I believe it is a good candidate for being published in PLOS Medicine.”

We appreciate your insightful suggestions which have helped further strengthen the paper.
