# Supplementary material for: Association of preterm birth with lipid disorders in early adulthood: A Swedish cohort study
Source: PLoS Med. 2019 Oct 18;16(10):e1002947. doi: 10.1371/journal.pmed.1002947 (PMC6799885; doi:10.1371/journal.pmed.1002947)
Supplement: Attachment — Submitted filename: Crump_Response_to_Reviewers_PLOS_Med_R3_v2.docx [file pmed.1002947.s005.docx]

September 9, 2019

Dr. Casey Crump

Icahn School of Medicine at Mount Sinai

One Gustave L. Levy Place, Box 1077

New York, NY 10029

RE: PMEDICINE-D-19-01959R2

Dear Editor:

Thank you again for your favorable review of our manuscript entitled “Association of Preterm Birth with Lipid Disorders in Early Adulthood: A Swedish Cohort Study”. We appreciate your additional suggestions for further enhancing the paper. We are now submitting a revised manuscript for your consideration after responding to each of those suggestions. Our detailed responses are listed below.

**EDITOR COMMENTS**

1) “Title – association with, instead of risk of”

*Response*: We agree and have changed the title accordingly (Title Page, lines 1-2).

2) “Abstract - Please add a sentence on the limitations of your study as the final sentence of the Methods and Findings section of the abstract. Around line 42, add "The main study limitations were ..." or similar”

*Response*: We have now added a limitations statement in the Abstract as suggested (Abstract, “Methods and Findings” section, lines 41-42).

3) “Around line 100 or line 150, state whether there was an analysis plan, and if so attach the document as a Supp file. If no analysis plan exists please state when the analyses were planned in relation to the data analysis.”

*Response*: The analytic approach was determined prior to beginning the data analyses, though there was no formal statistical analysis plan. We have now added a statement regarding this (Methods, “Other Study Variables” subsection, lines 149-151).

4) “Update reference 5, or please provide a copy of the acceptance letter”

*Response*: We have updated Reference 5 to include the correct citation from *JAMA Pediatrics*.

5) “Ref 34 has information that can be trimmed – please refer to style guide for refs.”

*Response*: We have now trimmed the unnecessary information from Reference 34.
